# Measuring Information Acquisition from Sensory Input Using Automated Scoring of Natural-Language Descriptions

**DOI:** 10.1371/journal.pone.0093251

**Published:** 2014-04-02

**Authors:** Daniel R. Saunders, Peter J. Bex, Dylan J. Rose, Russell L. Woods

**Affiliations:** Schepens Eye Research Institute, Massachusetts Eye and Ear, Boston, Massachusetts, United States of America; New York University, United States of America

## Abstract

Information acquisition, the gathering and interpretation of sensory information, is a basic function of mobile organisms. We describe a new method for measuring this ability in humans, using free-recall responses to sensory stimuli which are scored objectively using a “wisdom of crowds” approach. As an example, we demonstrate this metric using perception of video stimuli. Immediately after viewing a 30 s video clip, subjects responded to a prompt to give a short description of the clip in natural language. These responses were scored automatically by comparison to a dataset of responses to the same clip by normally-sighted viewers (the crowd). In this case, the normative dataset consisted of responses to 200 clips by 60 subjects who were stratified by age (range 22 to 85y) and viewed the clips in the lab, for 2,400 responses, and by 99 crowdsourced participants (age range 20 to 66y) who viewed clips in their Web browser, for 4,000 responses. We compared different algorithms for computing these similarities and found that a simple count of the words in common had the best performance. It correctly matched 75% of the lab-sourced and 95% of crowdsourced responses to their corresponding clips. We validated the measure by showing that when the amount of information in the clip was degraded using defocus lenses, the shared word score decreased across the five predetermined visual-acuity levels, demonstrating a dose-response effect (*N* = 15). This approach, of scoring open-ended immediate free recall of the stimulus, is applicable not only to video, but also to other situations where a measure of the information that is successfully acquired is desirable. Information acquired will be affected by stimulus quality, sensory ability, and cognitive processes, so our metric can be used to assess each of these components when the others are controlled.

## Introduction

Mobile organisms are continually gathering information about their environment, and acting on it. The information arrives via sensory organs, such as eyes and ears, and must then be processed to derive usable facts about the world. This process is limited not only by the fidelity of the information source, but also by the organism’s sensory and cognitive processing ability. Since these sources of error jointly determine the organism’s functional capabilities, there is a pressing need for performance metrics of information acquisition. Existing approaches have focused on each of these stages in isolation, e.g. with computational models of information quality [1]–[3], sensory function estimates [4], [5] and cognitive assessments [6], [7], without considering the overall ability of the individual. Here we present a method for assessing acquisition and processing of information in humans, using free-recall reportage and a “wisdom of crowds” [8] approach to objectively measure the valid content of that reportage. While our application assesses vision, and visual stimulus quality, the high level nature of the task means that the approach also assesses cognitive functioning. Therefore, while the approach does not inherently diagnose the source of any impairment in acquiring information from sensory input, it can be made more specific through elimination of possibilities using other tests.

This method could be used with high-level perception in any sensory modality, but we developed it in our search for an objective metric for video quality. In particular, as a method for assessing the benefit of image enhancement for people with vision loss. Modern digital image processing has the potential to modify images to make them more visible to people with vision impairment. It is increasingly feasible to apply such modifications to video in real time, which could improve the accessibility of television and movies. However, there is a lack of well-established, objective techniques for evaluating the potential benefit of image enhancement [9]. Apart from approaches that use computer models of the human visual system [10]–[13], the most common methods for estimating video quality are side-by-side comparisons of videos [14], [15], judgments of video quality on a rating scale [16]–[19], and observations of whether participants choose to set some level of enhancement rather than no enhancement when given the ability to adjust the amount of enhancement [17], [20].

A limitation of these methods is that they rely only on subjective impressions of video quality, and do not include direct assessment of whether an enhancement aids performance. In the domain of watching video for recreation (e.g. television), it is difficult to define what constitutes task performance. This, despite TV watching being an activity to which many adults devote a significant portion of their leisure time, find a benefit from as a source of relaxation, use as a way to spend time with other people, and learn from about the world and their community. A potential objective approach to assessing TV watching is to measure the quantity and accuracy of information that is acquired by the viewer while watching. Most aspects of vision (e.g. contrast sensitivity, colour vision, perception of fine detail, figure-ground segregation, and face recognition) are engaged when watching a TV program, and if any of these abilities are degraded, information acquisition will be affected. Similarly, degrading or enhancing (in an effective way) the video may also be able to affect information acquisition. Although people more often watch TV for enjoyment than specifically to acquire information (e.g. news), here we are considering the relevant information to be any content that needs to be understood to follow and appreciate the content of the video. That information could include the identity of the people on screen, facial expressions or the nature of the setting, without which the viewing experience may not be coherent or pleasurable. For a short video, much of the information that is acquired should be available to the viewer to report immediately after viewing.

In the related domain of reading comprehension, many studies have attempted to measure information acquisition from reading material, using multiple choice questions, replacement of deleted words, or immediate free recall [21], [22]. The free-recall approach, of having subjects describe what they have just read in their own words, has been advocated on the basis that it reflects correct grammatical interpretations [22], and that it is much less susceptible to guessing than multiple choice questions [23]. Natural-language responses are scored according to the amount of correct content from the passage, typically using a rubric created by the experimenters. Scores have been found to correlate with the stimulus quality, as manipulated by adding optical distortion to the image of the text [21]. In the context of eyewitness reportage of a short crime video, methods that included a free-recall component have been employed, with responses scored using a rubric developed by the experimenters that counted “attributes” in the response [24], [25].

Two studies that directly measured information acquisition in the domain of video used quizzes about the content of TV episodes developed from the corresponding Assistive Description scripts [26], [27]. In those studies, subjects answered two-choice questions about a 10-minute video segment. Though a small benefit of the video enhancement for the people with vision impairment was demonstrated, the authors noted that the baseline performance level of over 70% correct may have led to a ceiling effect. In another study on the effects of video quality on information acquisition, in the context of distance learning by video conferencing, quizzes did not detect any differences due to video degradation [28]. These results agree with the reading comprehension literature regarding the limited value of quizzes, due to lack of sensitivity and difficulty of construction. The subjective decisions that go into choosing quiz questions are also a concern.

Our solution to the need for objective and unbiased evaluation of the content of a natural-language response involves presentation of the stimulus to a suitable group of people to generate a reference dataset. A new response to that stimulus is then compared to these “normal” responses. The comparison is made using an objective approach that can be broadly categorized as computational linguistics, though the most successful algorithm that we have identified so far is surprisingly simple.

As an illustration of this new information acquisition metric, we describe its use for video stimuli: 30 s segments from Hollywood films and documentaries. In response to an open-ended prompt immediately after viewing, subjects gave a natural-language description of what they could recall about the video clip. Automated scoring algorithms were evaluated, and we report an experiment which validated the method using artificially degraded vision.

### Automated Scoring of Natural-language Descriptions

Free-recall methods for measuring information acquisition, such as those used in reading comprehension, typically score the responses using a manual coding system [29]–[31]. In one scoring system [31], coders compare the response to the original passage to count the number of concept words and concept-linking words they have in common, resulting in a final score in terms of “idea units”, which are similar to the “attributes” used when scoring crime video responses [24], [25]. However, these systems require trained coders, and take a great deal of time [32]. Bernhardt [29] estimated up to 10 minutes per response, while Heinz [33] found that manual scoring took 3 minutes per response. This is in addition to the time required to construct and validate rubrics for specific stimuli. There is always the risk that the observers have a different perception of the meaning of the stimulus than the experimenter constructing the rubric (e.g. factors such as gender, age or race). Finally, at least a subset of the responses, if not all, must be scored at least twice by different individuals to establish inter-rater reliability.

Therefore, rather than scoring the free-recall descriptions of the video clips manually using one of these systems, we developed an automated scoring strategy. Our algorithms compare responses to a normative dataset of responses to the same video clips. This approach is similar to one that has been used to evaluate machine translations [34], and to automatically grade student papers [35]. In those studies, responses were scored by comparison to at least one “gold standard” response, with greater similarity being taken to indicate higher quality content.

For most media clips, a gold standard is not available, and the development and validation of a gold standard would require significant experimental effort, while still being subject to bias towards the experimenters’ expectations. In the present study, we compare a response to many normative responses, to allow for a range of valid types of responses that are criterion-free. We establish a distribution of standard responses by collecting the natural language responses of a sample of unbiased viewers. In this distribution, some details will be mentioned by most viewers, whereas other details will be mentioned only by a minority of viewers. If a video modification, or an impairment of vision, leads to the acquisition of less information or to erroneous inferences, then the responses should be less similar to the response distribution from normally-sighted viewers for the same video clip, just as student papers that contain less correct knowledge will be scored lower using the comparison approach. When the normative dataset is sufficiently large, some concepts will be mentioned repeatedly, presumably because they are important, and this can be used to weight the scoring. Also, in a large normative dataset, less prominent features of the clip are more likely to be mentioned at least once, which gives a basis for recognizing these features in new responses and thereby avoiding penalizing less common responses.

For the purpose of this scoring method, we collected a large set of free-recall descriptions of the video clips from normally-sighted individuals who came into the lab (lab-sourced). There were 60 lab-sourced participants, with equal numbers of men and women in three equally sized age groups: under 60 years old, 60–70 y, and greater than 70 y. They all had binocular visual acuity better than or equal to 20/30, no ocular conditions in their self-reported ophthalmic history, and healthy retinas (assessed using fundus photography). We selected 200 video clips of 30 s duration from Hollywood films and TV programs, representing the genres Cartoon, Documentary/Nature, and Drama/Other (40, 40, and 120 clips respectively). Clips were presented with audio, but participants were instructed not to comment on it. Participants saw 40 clips each, leading to 2,400 responses in total, or 12 responses per video clip across all 60 participants. On completion of each video clip, the subject saw two prompts, “Describe this movie clip in a few sentences, as if to someone who has not seen it” and “Is there any other detail you want to mention?” The audio recordings of the two responses were concatenated to make a single response per video per participant. The responses were automatically transcribed using the speech recognition program MacSpeech Scribe (Nuance Communications, Burlington, MA, USA). We pilot tested the built-in speech recognition function of the Apple iPad 2 (Apple, Cupertino, CA, USA) for this purpose, but found it to be too limited both in its accuracy and its 90 s maximum recording time. These transcriptions were then corrected by workers on Amazon Mechanical Turk, 112 individuals in all, who listened to the audio and edited the text to match it. Although spot inspection suggested that the accuracy of spelling was high for these transcript editors, there were still occasional mistakes, such as confusing “dessert” and “desert”.

As described in detail in Saunders, Bex and Woods [36], we also collected a separate set of natural-language video descriptions from Amazon Mechanical Turk workers (crowdsourced), with the same prompt and the same video clips. In this dataset, there were 20 responses per video clip rather than 12, and the demographics of the sample (*N* = 99) were not controlled. However, we did collect self-reported demographic information, and the reported median age was 35 y (range 20–66 y), with 63% of the workers female.

### Comparison of Scoring Algorithms

We considered several algorithms for scoring the responses, all based on computing the similarity of responses to the normative responses to the same video clip, such that greater similarity corresponded to a better score. The evaluation used a take-one-out procedure: for each response in the normative dataset, we removed it from the dataset (e.g. 2,400), and scored it based on the remaining dataset (e.g. 2,399) as if it were a new response. These scores were used to classify the video clip from which the response originated, by determining which of the 200 clips had the highest average similarity between the new response and all the responses to that clip in the normative database. We then compared text-based similarity algorithms using the resulting percent correctly classified.

The text passage similarity metrics we considered were derived from computational linguistics. In all cases, we first processed the text with the Text to Matrix Generator toolbox for MATLAB [37], which, as a first step, removed a list of stop words from the text passage, including less informative words such as “of” and “the”, as well as verbal interjections such as “um” and “sorry”. The first approach to passage similarity that we evaluated was Latent Semantic Analysis [38]. LSA uses a pre-existing corpus of English text documents to construct a semantic space in which the distance between words reflects how often they co-occur in the same document (where documents correspond to responses in our method), or co-occur with words that themselves co-occur. For example, words such as “boat” and “anchor” will be drawn closer in semantic space because of their appearance in the same documents, while “boat” and “ship” will also be drawn closer, even if they do not co-occur in any document, because they are associated with other words that do co-occur. The corpus used was a combination of the freely available ukWaC [39] and Wackypedia [40] corpora, derived from the Web and Wikipedia, which together contain nearly three billion words. Passages were scored for semantic similarity based on their distance within the semantic space that was constructed.

The second approach that we tried was a similar vector space model (VSM) of semantics [41], where the co-occurrence of words in the corpus was judged not within documents but rather within a window of either 2 words or 20 words to the left and right of the target word. Subsequently, the semantic space was created in the same way, and the similarity scores were derived from the distance in the resulting semantic space. The third approach that we tested was the Distributional Memory model [42], which is also based on analyzing a corpus to determine the semantic relationship between words, but incorporates information about grammatical relations rather than only co-occurrence. It used a somewhat larger text corpus that included the British National Corpus.

Finally, for our fourth approach, we computed passage similarity by simply counting the number of words shared between two passages, after removal of stop words. Words that occurred more than once within a passage were only counted as a single match. Therefore, the score was the average of the vocabulary words shared with all the remaining responses to a video clip.

The highest rate of correct classification, that is, matching responses to the video clip of origin, was the simple count of shared words, for both datasets (Table 1). This result was surprising, because unlike the other algorithms, it does not have a mechanism for dealing with synonyms, such as “river” and “stream”. Since the strings do not match, they will not increase the similarity score between two passages. Nor does the shared word algorithm explicitly deal with word endings, for example considering “read” and “reading” to be two unrelated words, as well as “book” and “books”. However, with a large enough normative dataset, several synonyms for a concept will naturally occur among the responses, which increases the chances that a particular choice of words for a concept in a new response will be recognized. We suspect that while LSA and other algorithms deal with synonyms, they may have found false synonyms that contributed noise to the scores. Whatever the reason, the shared word count was the best of the algorithms that we tested, and achieved a classification rate of 75.4% for the lab-sourced dataset and 94.7% for the crowdsourced, as compared to the chance rate of 0.5% (i.e. 1/200 clips). When the lab-sourced dataset was used to classify crowdsourced responses using the shared word count, the rate decreased, to 82.5%, while when the crowdsourced dataset was used to classify lab-sourced responses, it improved the classification rate, to 81.0%. When a pooled dataset consisting of both the lab-sourced and crowdsourced responses was used to classify responses, the overall mean classification rate was 90.3%.

**Table 1:** Comparison of Performance of Text Similarity Algorithms.

Text similarity algorithm	Lab-sourced responses correctly classified (*N* = 2,400)	Crowdsourced responses correctly classified (*N* = 4,000)
Latent Semantic Analysis	33.6%	63.5%
VSM, 2-word window	42.8%	78.3%
VSM, 20-word window	37.8%	74.4%
Distributional memory	36.1%	70.7%
Shared words	75.4%	94.7%

Therefore, in the subsequent results we report the scores obtained by averaging the number of non-repeating words that appeared in both the target response and each of the normative responses for the corresponding video clip, after removal of stop words. If the same word appeared in many normative responses, it was effectively weighted more heavily, whereas multiple occurrences of the same word within a normative response did not affect the score.

### Importance of Normative Dataset Size

**Figure 1:**
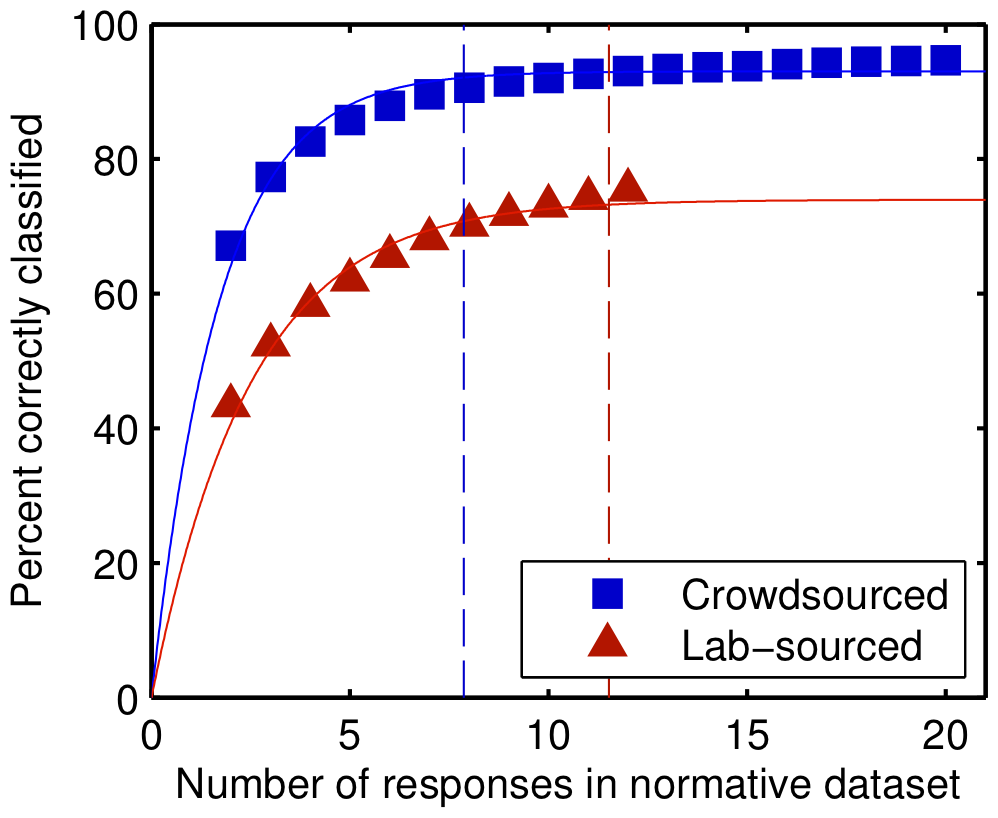
Response classification rate for smaller dataset sizes. Results of simulations on the two datasets for the percent correctly classified when the size of the normative dataset was reduced from its full size of either 20 or 12 responses per video clip. Each point is based on 100 random samples. The solid line indicates the best fit of a two-parameter exponential function, while the dashed line indicates the point at which the function reached 99% of the asymptote.

Would a smaller normative dataset be as effective? We computed the percent correctly classified for normative datasets with fewer than 12 responses per video clip (20 in the case of the crowdsourced dataset), by randomly sampling *n* responses for each video and recomputing the percent of responses that were correctly classified according to their originating video clip, again using a take-one-out strategy and the shared word scoring method (relative to the corresponding dataset). We repeated the randomization procedure 100 times for each value of *n*, with *n* ranging between 2 and the full number of responses available. Figure 1 shows how the percent correctly classified rapidly increased, until it slowed at the higher values of *n*. We fitted a two-parameter exponential function from which we determined the *n* at which the function reached 99% of its asymptotic value. The classification rate reached 99% of the asymptote at *n* = 7.9 for the crowdsourced dataset, and at *n* = 11.5 for the lab-sourced dataset.

**Figure 2:**
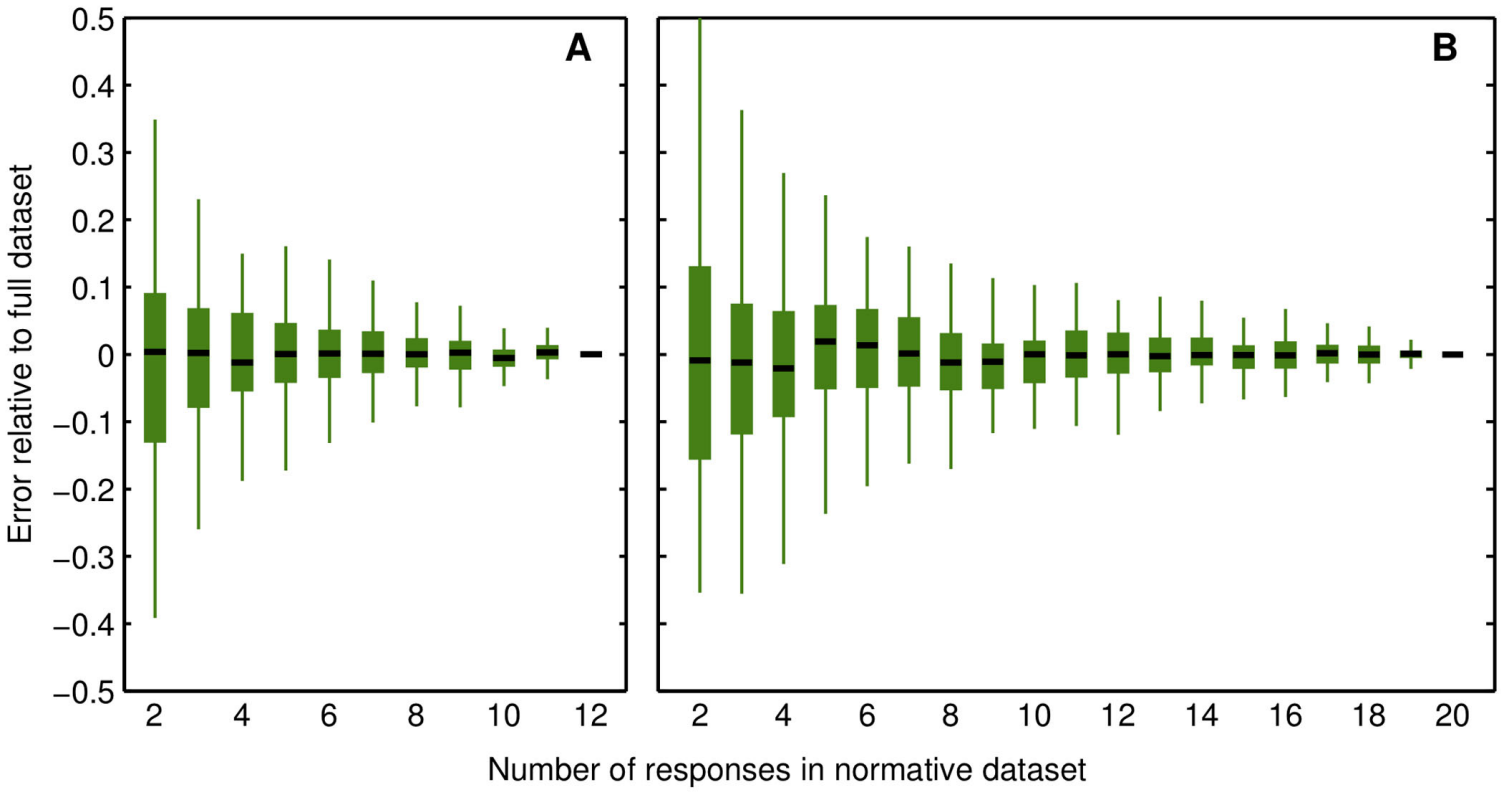
Error distribution of shared word scores for smaller dataset sizes. Error of the mean word score, relative to the score for the full normative dataset, as a function of the size of the A) lab-sourced dataset, or B) crowdsourced dataset. There were 100 random samples for each set size. Boxes indicate the interquartile range (IQR), the whiskers contain the range +/− 1.5 * IQR beyond the limits of the boxes (corresponding to 99.7% of the area of a normal distribution).

As another way to evaluate algorithm performance with smaller normative datasets, the error in the score of a particular response using a particular random subset was estimated as the difference between the computed score and the score with the full normative dataset. Figure 2 shows that there was not a systematic bias with a smaller normative dataset, but that the standard deviation of the error was larger. Depending on the application and the desired reliability, fewer than 12 responses per video clip in the normative data set might be feasible, but reliability drops quickly if the size is reduced by more than a few responses below that, particularly in the lab-sourced dataset.

### Effect of Reduced Visual Acuity on the Measure of Information Acquisition

We conducted an experiment to validate the average shared word score as a measure of information acquisition. To assess a dose response effect, participants wore defocus lenses of different powers to induce optical blur, while they viewed a subset of video clips and gave responses as in the normative data collection. We hypothesized that lower levels of visual acuity, induced by the defocus lenses, would produce lower shared word scores.

## Methods

This research was approved by the Massachusetts Eye and Ear Infirmary Human Subjects Committee. All participants in the experiment, and the normative dataset collection, gave informed, explicit consent (either with a signature or by clicking a box on a Web form).

We recruited 15 participants from the community with a median age of 34 y (21 to 67 y). They all had binocular visual acuity better than or equal to 20/20, no ocular conditions in their self-reported ophthalmic history, and healthy retinas. Participants had not contributed to the normative dataset, and so had not viewed the video clips previously. Using a visual acuity chart, we selected spherical defocus lenses for each participant that produced visual acuities of 20/20 or better, 20/50, 20/125, 20/320, and 20/800.

We selected 20 clips for testing from the set of 200 that were used in collecting the normative dataset, with each genre represented proportionally (4 Cartoon, 4 Documentary/Nature, and 12 Drama/Other). Each participant viewed all 20 clips in random order, with audio included, looking through defocus lenses that were switched in random order between each trial, for a total of 4 trials per visual acuity condition per participant. Participants received the same two prompts asking for a description of the movie as in the normative data collection, and their verbal responses were transcribed in the same manner using MacSpeech Scribe and Mechanical Turk workers.

Responses were scored by counting the average number of words in common with the 12 responses for the originating video in the lab-sourced normative dataset. We used a mixed-model analysis [43] to test for an effect of the fixed factor, “acuity condition”, since “participant” and “video clip” were fully-crossed random factors.

## Results and Discussion

**Figure 3:**
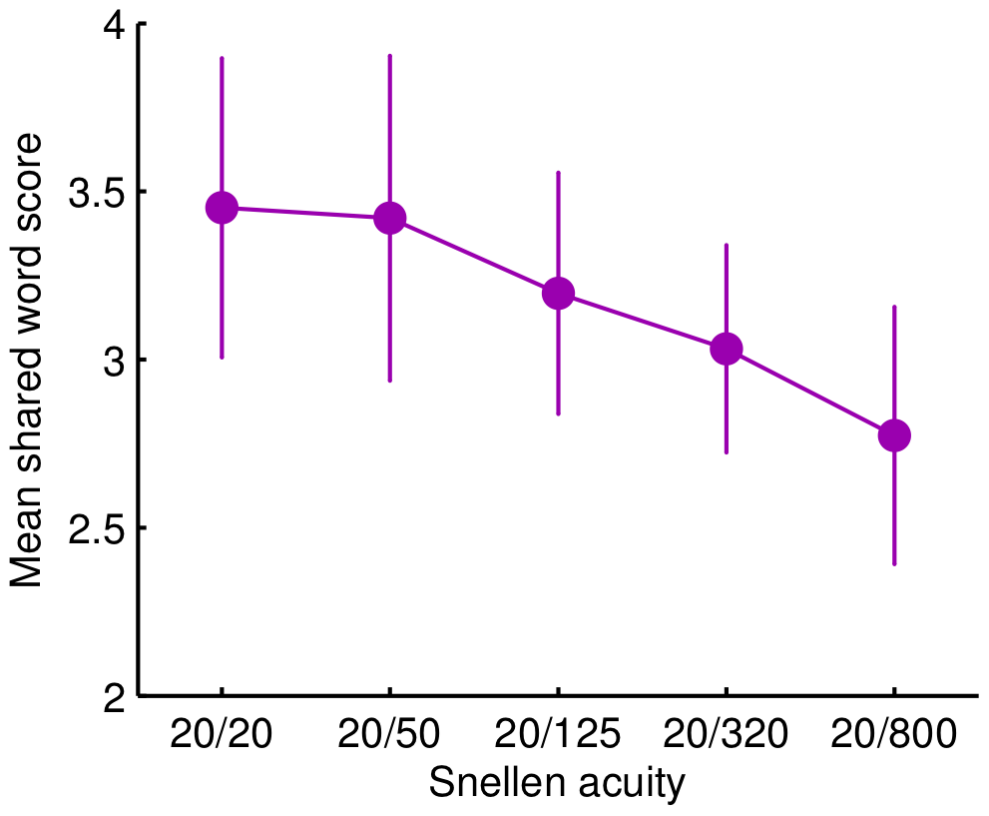
Effect of visual acuity on shared word score. Mean number of words shared with responses to the same clip in the lab-sourced dataset, for different levels of visual acuity as achieved with defocus lenses. Error bars indicate 95% confidence intervals.

Figure 3 illustrates the reduction in shared word score due to acuity condition, with a significant overall difference among the acuity levels, *p*<0.001. Comparing all conditions to the 20/20 acuity condition, the 20/800 condition produced a significantly lower average score, *p*<0.001, and so did the 20/320 condition, *p* = 0.007. The shared word scores in the other acuity conditions were not significantly different from the 20/20 condition. There was also a significant decrease in mean score between the 20/50 condition and the 20/320 condition, *p* = 0.012. As another test of the dose response effect, the trend for a reduction in shared words as visual acuity reduced was significant (Spearman’s rho = −0.17, *p* = 0.003), even when not correcting for video clip or participant differences.

The shared word measure was therefore capable of detecting an effect of lowered visual acuity with 60 responses per acuity condition. Impairing vision with defocus lenses significantly reduced the amount of information that the viewer could access in the video clips. This demonstration provides support for the proposal that shared word scores from natural-language descriptions are a valid and reliable measure of information acquisition from a video clip.

## General Discussion

In this paper, we describe a novel approach to evaluating information acquisition, that does not rely on subjective judgments or manual coding of responses, and apply it to watching TV and movies for recreation. Since the scoring is objective, it is not vulnerable to experimenter bias. While the process of information acquisition from video is complicated, involving many stages of cognitive and perceptual function, when there is less information from the image available, as in the defocus experiment, less information can be acquired. Therefore declining scores for the natural-language descriptions associated with lower acuity is a necessary outcome for any valid measure of information acquisition.

This method requires a substantial normative dataset, consisting of multiple natural-language descriptions of each stimulus, with, ideally, a large number of stimuli that should represent different characteristics of the stimuli of interest. We showed previously that crowdsourcing using Mechanical Turk is an efficient and low cost way to collect such a natural-language dataset, with properties similar to those of a dataset collected under more controlled conditions in the lab [36]. That study also explored ways in which the two datasets were different, which can explain the difference in mean classification rates: the lab-sourced participants had a more varied vocabulary, and wrote shorter responses on average. They were also more likely to be male, and were older on average, both factors that were found to be associated with lower shared word scores. Here, we demonstrated through resampling that datasets smaller than those we collected may also be effective. With the lab-sourced data collection method there may be little gain in precision beyond 12 responses per stimulus. The analysis also suggests that with the somewhat more homogenous results of crowdsourced data collection, as few as 8 responses per stimulus makes an effective normative dataset for scoring responses drawn from the same population.

The concept of measuring information acquisition through free recall has applications outside of video viewing and reading comprehension. For example, a similar protocol could be used to test information acquisition from auditory stimuli, which could then be used to identify differences in hearing ability, or to evaluate sound compression algorithms. The measure could also suggest the presence of aphasias or other forms of disfluency in speech or writing, when standard vision test scores are within the normal range. Alternatively, if both speech and low-level vision are normal, a low score could suggest cognitive impairments, such as those resulting from traumatic brain injury or Alzheimer’s disease. A deficit in any of the stages of processing from the eye to the natural-language response would lower the score, and the contribution of other standardized tests could isolate which stage, or combination of stages, is causing the poor performance.

Improvements to the automatic scoring algorithm could increase the method’s sensitivity. In the present study, it could not detect the smallest differences in visual acuity, for example between 20/20 and 20/50, with the sample size that was used. It seems that at 20/50, most of the salient and frequently-mentioned details of a video clip were still acquired. Therefore, there are limitations to the sensitivity of the method in its present form for monitoring and diagnosis of perceptual and cognitive problems in individuals. It is possible that the inclusion of audio may have reduced the sensitivity of the method to changes in visual acuity, even though participants were instructed to ignore the sound track. Another obstacle to interpreting scores for individuals is the possibility of subjects who use a distinctive vocabulary, or interpret the video clip in an idiosyncratic manner. These scores will be systematically lower due to divergence of their responses from the normative responses. The same problem affects groups of subjects who are not native speakers of the language of the normative dataset. However, although we evaluated several of the more popular algorithms for passage similarity and found them less effective than a simple count of shared words, more sophisticated algorithms for comparing responses to the normative dataset may be able to better detect individual differences in vision or video quality. In any case, we have demonstrated via the defocus experiment that even the simplest word matching method can detect differences due to within-subject experimental manipulations. Therefore, it can also be used to assess treatment effectiveness within individuals, and to track the progression or remediation of a condition.

Organisms with healthy vision and an unimpaired view of their environment can extract a wealth of information about it, starting from raw sensory input. We have presented a novel method for quantitatively assessing this general information acquisition in humans, that is objective and requires relatively few experimenter resources.

## References

[1] Wang Z, Bovik AC, Sheikh HR, Simoncelli EP (2004) Image quality assessment: From error visibility to structural similarity. IEEE Transactions on Image Processing 13: 600–612. 15376593 10.1109/tip.2003.819861

[2] Stockham Jr TG (1972) Image processing in the context of a visual model. Proceedings of the IEEE 60: 828–842.

[3] Jepsen ML, Ewert SD, Dau T (2008) A computational model of human auditory signal processing and perception. The Journal of the Acoustical Society of America 124: 422. 18646987 10.1121/1.2924135

[4] Bailey IL, Lovie J (1976) New design principles for visual acuity letter charts. American Journal of Optometry and Physiological Optics 53: 740–745. 998716 10.1097/00006324-197611000-00006

[5] Sekuler R, Tynan P (1977) Rapid measurement of contrast-sensitivity functions. American Journal of Optometry and Physiological Optics 54: 573. 596406 10.1097/00006324-197708000-00014

[6] Nasreddine ZS, Phillips NA, Bédirian V, Charbonneau S, Whitehead V, et al (2005) The Montreal Cognitive Assessment, MoCA: A brief screening tool for mild cognitive impairment. Journal of the American Geriatrics Society 53: 695–699. 15817019 10.1111/j.1532-5415.2005.53221.x

[7] Crawford JR, Parker DM, McKinlay WW (1992) A Handbook of Neuropsychological Assessment. Exeter, UK: Lawrence Erlbaum Associates.

[8] Surowiecki J (2004) The Wisdom of Crowds: Why the Many Are Smarter Than the Few and How Collective Wisdom Shapes Business, Economies, Societies and Nation. New York, NY: Doubleday.

[9] Peli E, Woods RL (2009) Image enhancement for impaired vision: The challenge of evaluation. International Journal of Artificial Intelligence Tools 18: 415–438. 20161188 10.1142/S0218213009000214 PMC2727758

[10] Wang Z, Sheikh HR, Bovik AC (2003) Objective video quality assessment. In: Furht B, Marques O, editors. The Handbook of Video Databases: Design and Applications. Boca Raton, FL: CRC Press. 1041–1078.

[11] Barten PGJ (1990) Evaluation of subjective image quality with the square-root integral method. Journal of the Optical Society of America A: Optics, Image Science and Vision 7: 2024–2031.

[12] Eskicioglu AM, Fisher PS (1995) Image quality measures and their performance. IEEE Transactions on Communications 43: 2959–2965.

[13] Lubin J (1995) A visual discrimination model for imaging system design and evaluation. In: Peli E, editor. Vision Models for Target Detection. Singapore: World Scientific. 245–283.

[14] Kim J, Vora A, Peli E (2004) MPEG-based image enhancement for the visually impaired. Optical Engineering 43: 1318–1328.

[15] Satgunam P, Woods RL, Bronstad MP, Peli E (2010) Factors affecting image quality preferences. SID Symposium Digest of Technical Papers: Blackwell Publishing Ltd. 94–97.

[16] Hamberg R, Ridder H (1995) Continuous assessment of perceptual image quality. JOSA A 12: 2573–2577. 7500220 10.1364/josaa.12.002573

[17] Peli E (1999) Perceived quality of video enhanced for the visually impaired. Vision Science and Its Applications. Santa Fe, NM: OSA. 46–48.

[18] Pinson M, Wolf S (2003) Comparing subjective video quality testing methodologies. In: Ebrahimi T, editor. Visual Communications and Image Processing 2003: International Society for Optics and Photonics. 573–582.

[19] Peli E (2005) Recognition performance and perceived quality of video enhanced for the visually impaired. Ophthalmic and Physiological Optics 25: 543–555. 16343130 10.1111/j.1475-1313.2005.00340.x PMC1343473

[20] Fullerton M, Woods RL, Vera-Diaz FA, Peli E (2007) Measuring perceived video quality of MPEG enhancement by people with impaired vision. Journal of the Optical Society of America (A) 24: B174–B187. 10.1364/josaa.24.00b174 PMC2131737 18059909

[21] Dickinson CM, Rabbitt PMA (1991) Simulated visual impairment: Effects on text comprehension and reading speed. Clinical Vision Sciences 6: 301–308.

[22] Bernhardt EB (1983) Testing foreign language reading comprehension: The immediate recall protocol. Die Unterrichtspraxis/Teaching German 16: 27–33.

[23] Pyrczak F (1975) Passage-dependence of reading comprehension questions: Examples. Journal of reading 18: 308–311.

[24] Allwood CM, Innes-Ker AH, Homgren J, Fredin G (2008) Children’s and adults’ realism in their event-recall confidence in responses to free recall and focused questions. Psychology, Crime and Law 14: 529–547.

[25] Allwood CM, Ask K, Granhag PrA (2005) The Cognitive Interview: Effects on the realism in witnesses’ confidence in their free recall. Psychology, Crime & Law 11: 183–198.

[26] Fine EM, Peli E, Brady N (1996) Evaluating video enhancement for visually impaired viewers. Vision ’96: Proceedings of the International Conference on Low Vision. Madrid, Spain: ONCE. 85–92.

[27] Peli E, Fine EM, Labianca AT (1996) Evaluating visual information provided by audio description. Journal of Visual Impairment and Blindness 90: 378–385.

[28] Kies JK, Williges RC, Rosson MB (1997) Evaluating desktop video conferencing for distance learning. Computers & Education 28: 79–91.

[29] Bernhardt EB (1991) Reading development in a second language: Theoretical, empirical, and classroom perspectives. Norwood, New Jersey: Ablex Publishing Corporation.

[30] Kintsch W (1974) The Representation of Meaning in Memory. Hillsdale, N.J: Erlbaum.

[31] Meyer BJF, Brandt DM, Bluth GJ (1980) Use of top-level structure in text: Key for reading comprehension of ninth-grade students. Reading Research Quarterly 16: 72–103.

[32] Alderson JC (2000) Assessing reading. Cambridge: Cambridge University Press.

[33] Heinz PJ (2004) Towards enhanced second language reading comprehension assessment: Computerized versus manual scoring of written recall protocols. Reading in a foreign language 16: 97–124.

[34] Papineni K, Roukos S, Ward T, Zhu W (2002) BLEU: A method for automatic evaluation of machine translation. Proceedings of the 40th Annual Meeting of the Association for Computational Linguistics. Philadelphia, PA: Association for Computational Linguistics. 311–318.

[35] Foltz PW, Wells AD (1999) Automatically deriving readers’ knowledge structures from texts. Behaviour Research Methods, Instruments and Computers 31: 208–214. 10.3758/bf03207712 10495802

[36] Saunders DR, Bex PJ, Woods RL (2013) Crowdsourcing a normative natural language dataset: A comparison of Mechanical Turk and in-lab data collection. Journal of Medical Internet Research 15: e100. 23689038 10.2196/jmir.2620 PMC3668615

[37] Zeimpekis D, Gallopoulos E (2005) Design of a MATLAB toolbox for term-document matrix generation. In: Dhillon IS, Kogan J, Ghosh J, editors. Proceedings of the Workshop on Clustering High Dimensional Data. Newport Beach, CA: SIAM. 38–48.

[38] Landauer TK, Dumais ST (1997) A solution to Plato’s problem: The latent semantic analysis theory of acquisition, induction, and representation of knowledge. Psychological Review 104: 211–240.

[39] Ferraresi A, Zanchetta E, Baroni M, Bernardini S (2008) Introducing and evaluating ukWaC, a very large web-derived corpus of english. In: Marrakech, Morocco: LREC Evert S, Kilgarriff A, Sharoff S, editors. Proceedings of the 4th Web as Corpus Workshop (WAC-4). 2008: 47–54.

[40] Baroni M, Bernardini S, Ferraresi A, Zanchetta E (2009) The WaCky wide web: A collection of very large linguistically processed web-crawled corpora. Language Resources and Evaluation 43: 209–226.

[41] Turney PD, Pantel P (2010) From frequency to meaning: Vector space models of semantics. Journal of Artificial Intelligence Research 37: 141–188.

[42] Baroni M, Lenci A (2010) Distributional memory: A general framework for corpus-based semantics. Computational Linguistics 36: 673–721.

[43] Janssen D (2011) Twice random, once mixed: Applying mixed models to simultaneously analyze random effects of language and participants. Behavior Research Methods 44: 232–247. 10.3758/s13428-011-0145-1 21858733

